# Distal pancreatectomy with celiac axis resection (DP-CAR) and vascular reconstruction for locally advanced pancreatic adenocarcinoma: A case report

**DOI:** 10.1016/j.ijscr.2025.111264

**Published:** 2025-04-04

**Authors:** Bagdaulet Iliyas, Yerik Nurlanbaev, Madiyar Nagasbekov, Shokan Kaniyev, Bolatbek Baimakhanov, Ildar Fakhradiyev

**Affiliations:** a Syzganov National Scientific Center of Surgery, Almaty, Republic of Kazakhstan; b S.D. Asfendiyarov Kazakh National Medical University, Almaty, Republic of Kazakhstan; c College of Medicine, Korea University, Seoul, South Korea

**Keywords:** Pancreatic ductal adenocarcinoma, DP-CAR, Celiac trunk prosthesis, Neoadjuvant chemotherapy, Vascular reconstruction, Pancreatic surgery, Anatomical variation, Case report

## Abstract

**Introduction:**

Pancreatic ductal adenocarcinoma (PDAC) is a highly aggressive malignancy that often presents at an advanced stage, especially when tumors involve the body and tail of the pancreas.

**Case presentation:**

This case report describes a comprehensive treatment approach for a 40-year-old female with locally advanced PDAC of the pancreatic body, characterized by invasion into the celiac trunk and splenic artery. Initial management included nine courses of FOLFIRINOX chemotherapy and 27 sessions of radiotherapy, resulting in significant tumor regression. Subsequently, a complex surgical procedure involving corporocaudal resection of the pancreas, splenectomy, and celiac trunk prosthetic reconstruction using an autograft from the great saphenous vein was performed.

**Discussion:**

Preoperative CT imaging revealed a rare anatomical variation where the celiac trunk and superior mesenteric artery shared a common origin from the aorta, while the left gastric artery branched separately, facilitating postoperative gastric perfusion. Postoperative recovery was uneventful, with liver enzyme levels stabilizing within normal limits. Follow-up imaging six months after surgery confirmed the absence of disease recurrence and the maintained integrity of the vascular reconstruction.

**Conclusion:**

This case highlights the importance of individualized treatment planning, the use of neoadjuvant therapy, and advanced surgical techniques to achieve favorable outcomes in patients with complex PDAC presentations.

## Highlights

• Modern technologies and advancements in diagnostic and surgical planning enable the successful execution of technically complex pancreatic operations, even in cases with body and tail invasion.
• DP-CAR with celiac trunk prosthetic reconstruction proved to be effective and safe, as evidenced by the uneventful postoperative course and the absence of disease recurrence within six months after surgery.
• Multimodal treatment, including chemotherapy combined with surgery, is also a crucial component of successful management.

## 1 Introduction

Pancreatic ductal adenocarcinoma (PDAC) is the most common malignant neoplasm of the pancreas, accounting for more than 90 % of all primary pancreatic tumors [1]. The disease is often diagnosed at advanced stages, particularly when the body and tail of the pancreas are affected, as symptoms are typically absent until the tumor begins to invade major blood vessels and nerve plexuses [2].

Consequently, only a limited number of pancreatic body tumors are resectable at the time of diagnosis, with vascular invasion and distant metastases remaining the primary factors limiting surgical intervention [3].

Approximately 30 % of patients are diagnosed with locally advanced PDAC, characterized by extensive vascular involvement but no signs of distant metastases [1]. For this patient group, distal pancreatectomy with celiac axis resection (DP-CAR) represents a crucial surgical strategy to achieve R0 resection, a critical determinant for improved survival. However, the success of this procedure largely depends on meticulous planning, including the preservation of hepatic and gastric arterial blood flow post-resection [4].

Candidates for DP-CAR typically undergo neoadjuvant chemotherapy with the FOLFIRINOX regimen, which helps reduce tumor size and increase the likelihood of successful resection. Studies have shown that patients receiving FOLFIRINOX chemotherapy prior to surgery have improved survival rates [5]. Despite the high risks associated with vascular invasion, modern surgical techniques, including the use of vascular grafts, enable successful operations with acceptable postoperative recovery outcomes [6].

The present clinical case illustrates a comprehensive approach to treating a patient with locally advanced pancreatic body cancer, involving neoadjuvant therapy and surgical resection with celiac axis prosthesis, demonstrating favorable outcomes even in anatomically complex scenarios.

## 2 Case report

This case report has been reported in line with the SCARE criteria [7]. Patient K., a 40-year-old female, was admitted to our clinic with complaints of epigastric discomfort, pain in the left hypochondrium, and general weakness. According to her medical history, in April 2022, a diagnosis of a pancreatic body tumor with invasion into the celiac trunk and splenic artery was first established based on a computed tomography (CT) scan of the abdominal organs. In July 2022, a laparotomy with a biopsy of the pancreatic tumor was performed. Histological analysis revealed a well-differentiated adenocarcinoma (G1) of the pancreas.

**Fig. 1:**
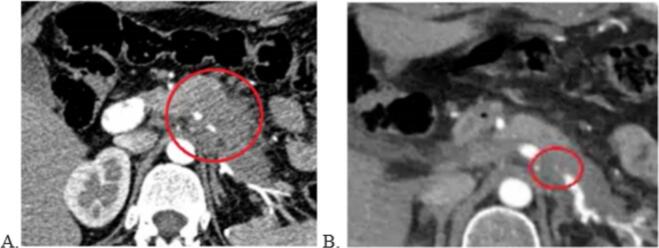
Contrast-enhanced computed tomography (CT) of the abdominal cavity. A) CT of the abdominal organs before chemotherapy, 26.04.2022. B) CT of the abdominal organs after completion of chemotherapy, 21.02.2023.

Following the confirmation of malignant cells, the patient underwent 9 courses of chemotherapy according to the FOLFIRINOX regimen and 27 sessions of radiotherapy from July to December 2022. Follow-up imaging demonstrated no signs of local tumor progression or distant metastases on a CT scan of the abdominal organs (Fig. 1).

In May 2023, considering the positive response to chemotherapy, surgical intervention was performed, consisting of a laparotomy, corporocaudal resection of the pancreas with splenectomy, and resection and prosthesis of the celiac trunk.

**Fig. 2:**
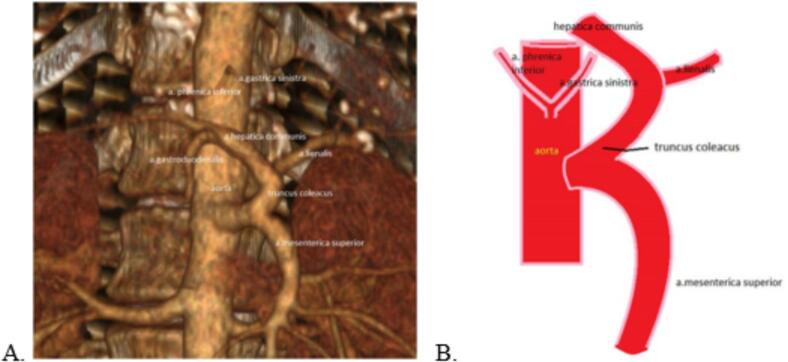
Anatomical Variation of the Celiac Trunk and Superior Mesenteric Artery Branching. A) Preoperative CT scan of the abdominal cavity showing a three-dimensional arterial reconstruction, illustrating the rare anatomical variation with the celiac trunk and superior mesenteric artery arising from the abdominal aorta as a common trunk. B) Schematic diagram depicting the variation in the branching pattern, where the left gastric artery originates separately from the abdominal aorta.

A distinguishing feature of this case was a rare anatomical variation in the branching of vessels from the abdominal aorta: the celiac trunk and superior mesenteric artery emerged from the aorta as a common trunk, while the left gastric artery originated separately from the aorta (Fig. 2).

**Fig. 3:**
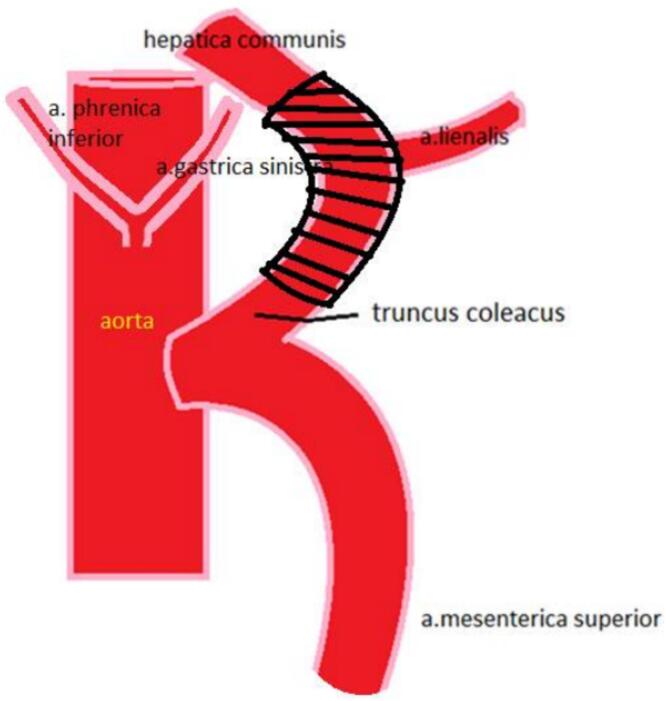
Schematic diagram of the resected section of the celiac trunk.

**Fig. 4:**
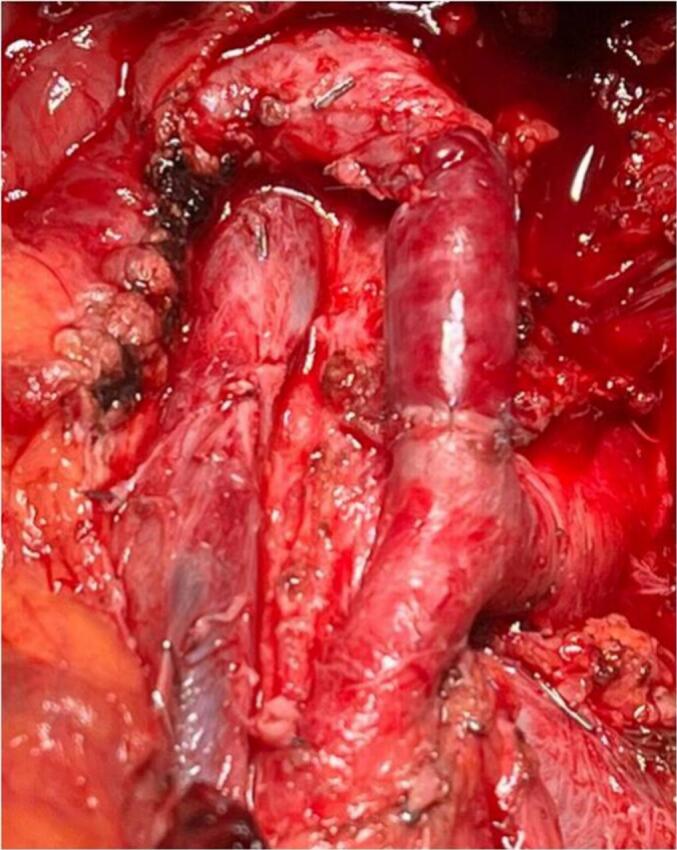
Intraoperative image showing the prosthesis of the celiac trunk using an autograft from the great saphenous vein.

Upon exploration of the abdominal cavity, a dense tumor mass measuring 5.0 × 3.0 cm was identified in the region of the pancreatic body and tail, with invasion into the superior mesenteric artery trunk and splenic artery. Due to the common origin of the celiac trunk and superior mesenteric artery from the aorta, a classical DP-CAR resection could have resulted in hepatic ischemia. Therefore, a decision was made to resect the trunk and subsequently reconstruct it with a prosthesis. An autograft from the great saphenous vein was used for this purpose (Fig. 3, Fig. 4).

This approach allowed for the restoration of normal blood flow, contributing to a successful postoperative recovery. Histological analysis showed moderately differentiated ductal adenocarcinoma of the pancreatic tail and body with lymphovascular and perineural invasion, classified as low-grade malignancy (G2).

**Fig. 5:**
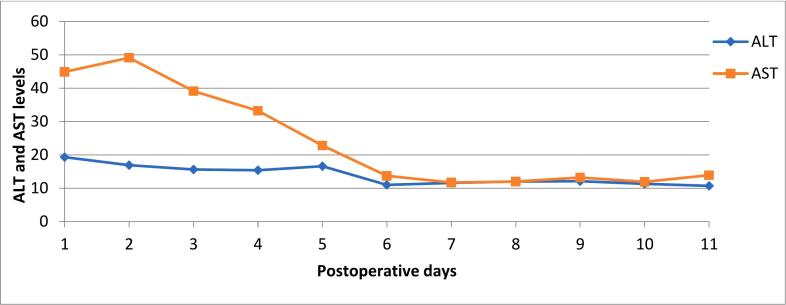
Post-surgery ALT and AST levels.

After surgery, the ALT and AST levels (Fig. 5) were monitored over an 11-day period. Initially, ALT levels remained relatively stable, ranging from approximately 18 to 20 units per liter, with a slight decrease observed on 6th postoperative day. In contrast, AST levels were markedly higher at the beginning, starting around 50 units per liter on first postoperative days.

AST showed a progressive decline over the subsequent days, dropping sharply to near 20 units per liter on 6th postoperative day. On 7th day after surgery, both ALT and AST levels had stabilized and remained consistent, with minor fluctuations, staying around or slightly below 20 units per liter through 11th postoperative day. This trend indicates a significant decrease in AST levels post-surgery, while ALT levels maintained a stable, normal range throughout the observation period.

**Fig. 6:**
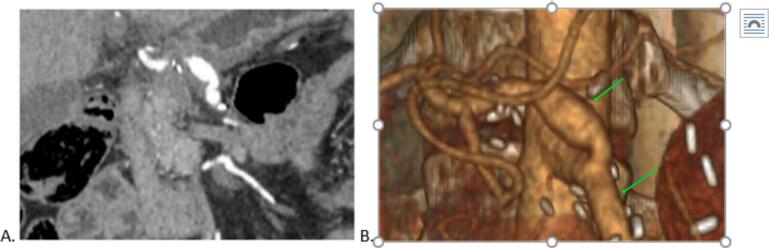
CT scan of the abdominal cavity six months post-surgery. A) Coronal projection showing the abdominal cavity in detail, indicating the absence of disease recurrence or pathological changes. B) Three-dimensional arterial reconstruction illustrating the vascular anatomy, with labels indicating: 1 – the prosthetic section of the celiac trunk; 2 – the superior mesenteric artery, both demonstrating maintained vascular integrity and normal blood flow.

The patient was discharged on the 12th postoperative day in satisfactory condition. Subsequently, she received three additional courses of chemotherapy according to the FOLFIRINOX regimen during the second and third months post-surgery. A follow-up CT scan of the abdominal organs six months later showed no signs of disease recurrence or distant metastases and confirmed the integrity of the reconstructed celiac trunk (Fig. 6).

## 3 Discussion

The Appleby operation (en bloc resection of the celiac trunk, distal pancreatectomy with splenectomy, and total gastrectomy) was first performed in 1953 to achieve more complete resection of lymph nodes in the parietal trunk for locally advanced gastric cancer. In 1976, Nimura adapted this technique for the resection of adenocarcinoma of the pancreatic body and tail [8].

This procedure, commonly referred to as the modified Appleby procedure when applied to pancreatic tumors, has demonstrated improved survival rates for patients with ductal adenocarcinoma of the pancreatic body and tail, particularly when an R0 resection can be achieved. It has also provided complete relief from severe pain caused by tumor invasion into the paravertebral plexuses [9]. Furthermore, larger case series have underscored its potential benefits. For instance, Truty et al. reported that in highly selected patients with locally advanced pancreatic body and tail adenocarcinomas, the modified Appleby procedure can result in favorable perioperative and long-term outcomes, highlighting the significance of patient selection, meticulous surgical planning, and a multidisciplinary approach [10].

In complex situations where the tumor involves critical vascular structures, a combination of advanced diagnostic imaging, neoadjuvant therapy, and surgical innovation is crucial. CT remains the most effective modality for staging pancreatic ductal adenocarcinoma, with reported sensitivity and specificity of up to 90 %, along with comparable accuracy in assessing arterial lesions [11]. The evaluation of arterial and venous involvement in the early arterial, pancreatic, and portal phases allows surgeons to precisely plan resections and potential vascular reconstructions.

Anatomical variations in the origin and branching pattern of the celiac trunk and superior mesenteric artery (SMA) further complicate surgical strategies [12]. In the present case, a rare configuration was identified where the celiac trunk and SMA originated as a common trunk, with the left gastric artery arising separately from the aorta. This vascular arrangement facilitated adequate blood flow to the stomach, reducing the risk of postoperative ischemia—one of the most severe complications associated with distal pancreatectomy and celiac axis resection (DP-CAR) [13,14]. Preoperative hepatic artery embolization can be considered to enhance collateral circulation and lower the risk of ischemia to the liver, but its efficacy remains controversial. In our patient, no such intervention was performed; instead, hepatic perfusion was monitored using laboratory data and imaging, with the transaminase levels now expressed by postoperative day (Fig. 5). We have also included a more detailed, graphical representation of these levels for clarity.

Another frequent postoperative complication, reported in approximately 30 % of patients undergoing standard distal pancreatectomy or DP-CAR, is external pancreatic fistula [15,16]. In our patient, close clinical and laboratory monitoring throughout the postoperative course revealed no such complications, and recovery was uneventful.

Overall, this case emphasizes the importance of a tailored, multimodal approach to treating locally advanced PDAC involving the body and tail of the pancreas. A combination of effective neoadjuvant therapy and technically complex surgery, including vascular resection and reconstruction, can provide an opportunity for R0 resection even in anatomically challenging scenarios. As our experience and published case series—including those by Truty et al.—demonstrate, achieving long-term survival in these patients hinges on thorough preoperative assessment, precise surgical execution, and vigilant postoperative follow-up.

## 4 Conclusion

Modern technologies and advancements in diagnostic and surgical planning enable the successful execution of technically complex pancreatic operations, even in cases with body and tail invasion. The experience of surgeons, the use of advanced diagnostic methods, and meticulous surgical planning contribute to favorable postoperative outcomes. Multimodal treatment, including chemotherapy combined with surgery, is also a crucial component of successful management. In our case, DP-CAR with celiac trunk prosthetic reconstruction proved to be effective and safe, as evidenced by the uneventful postoperative course and the absence of disease recurrence within six months after surgery.

## Consent

Written informed consent was obtained from the patient for publication and any accompanying images.

## Ethical approval

The study was approved by the Syzganov National Scientific Center of Surgery Local Ethics Committee, Almaty, Kazakhstan (protocol of the Local Ethics Committee No -5 (2022).

## Guarantor

Ildar Fakhradiyev

## Funding

No financial or nonfinancial benefits have been received or will be received from any party related directly or indirectly to the subject of this article.

## Author contribution

Baghdaulet Iliyas, Yerik Nurlanbaev, Madiyar Nagasbekov, Ildar Fakhradiyev: paper writing and editing. Ingkar Omarkyzy, Shokan Kaniev, Bolatbek Baimakhanov: literature review, supervision. Madiyar Nagasbekov, Ildar Fakhradiyev: Manuscript editing, picture editing

## Conflict of interest statement

The authors declare that they have no competing interests relevant to the content of this article.
